# Using generative AI to transform peptide hits into small molecule leads

**DOI:** 10.3762/bjoc.22.51

**Published:** 2026-04-30

**Authors:** Joshua Mills, Yu Heng Lau

**Affiliations:** 1 School of Chemistry, The University of Sydney, Camperdown NSW 2006, Australia; 2 ARC Centre for Innovations in Peptide and Protein Science, The University of Sydney, Australia

**Keywords:** diffusion models, drug discovery, generative AI, peptides, small molecules

## Abstract

There is a wealth of structural data on peptides that bind potential drug targets, serving as rich inspiration for designing small molecule inhibitors which can recapitulate key binding interactions while improving pharmacokinetic properties. With the rapid advancement of artificial intelligence capabilities including powerful generative models, there is significant potential for new AI-based tools to expedite the structure-based transformation of peptide hits into small molecule leads. In this Perspective, we highlight how AI-enabled prediction and design tools can potentially span the entire workflow from peptide to small molecule: target protein structure prediction, de novo peptide binder generation, diffusion models for generating novel small molecule scaffolds, and deep-learning predictors of binding affinity to rapidly triage candidates.

## Introduction

In drug discovery, peptides serve as accessible starting points for designing molecules that bind and inhibit a chosen protein target. Many enzyme targets (e.g., proteases) natively process peptides as their substrates. Peptide domains are also common motifs in protein–protein interactions, mediating the binding between protein partners that form functional complexes [1–3]. Furthermore, powerful high-throughput library screening technologies such as mRNA and phage display have revolutionised our ability to identify novel peptide binders, frequently generating binders with nanomolar affinity directly from screening campaigns [4–6].

While there has been increasing recognition of the translational potential of peptides in drug discovery, significant optimisation is typically required to convert a naïve peptide into a bona fide drug candidate [7]. Many promising peptide hits fail to translate beyond academic research, hampered by pharmacokinetic liabilities such as poor metabolic stability, limited membrane permeability, and low oral bioavailability. In some cases, peptides may be suitable for therapeutic use after installing a small number of chemical modifications (e.g., lipidation of peptide hormones such as GLP-1 receptor agonists). In other cases, however, challenging medicinal chemistry is required to achieve clinical efficacy, as exemplified by the complex development of the heavily modified tricyclic peptide MK-0616, a PCSK9 inhibitor in phase III clinical trials that was originally derived from an mRNA display screen of monocyclic peptides [8].

As an alternative to optimising peptide-based molecules, knowledge of key binding interactions exhibited by potent peptide binders can be leveraged to inform small-molecule design. The field of peptidomimetics seeks to design small molecules that mimic the binding mode of a peptide, while mitigating the pharmacokinetic liabilities associated with unmodified peptides. This peptide-first approach to drug discovery is particularly valuable for challenging targets such as protein–protein interactions, where traditional small molecule high-throughput screening often fails to yield hits [9]. While the term peptidomimetics has been used to describe a variety of molecular classes, including modified peptides and molecular scaffolds for display of amino acid side-chains, this Perspective focuses on non-peptidic small molecules with the appropriate shape and functionality to recapitulate the pharmacophore of the original peptide (Class D according to the classification proposed by Grossmann and co-workers [10]).

The challenge of transforming a peptide binder into a small molecule mimic is not new [11]. Indeed, there are well-known historical examples of blockbuster drugs derived from native peptide substrates. A classic example is the ACE inhibitor captopril, an analogue of a snake venom peptide, the development of which has been cited as an early success story for structure-based rational drug design [12–13]. Despite the long history, there is still no straightforward and generalisable workflow with a reasonable success rate for achieving the transformation from peptide to small molecule inhibitor.

With the rapid maturation of computational tools based on artificial intelligence (AI), this Perspective highlights selected nascent examples where AI has been used for small molecule design by leveraging data on peptide binders, and proposes potential opportunities where generative AI and machine learning (ML) tools may augment various stages throughout the pipeline from peptide hit discovery to small molecule lead.

## Perspective

### Current workflows for designing small molecules that mimic peptide pharmacophores

#### Illustrative example of a non-AI workflow

**Figure 1:**
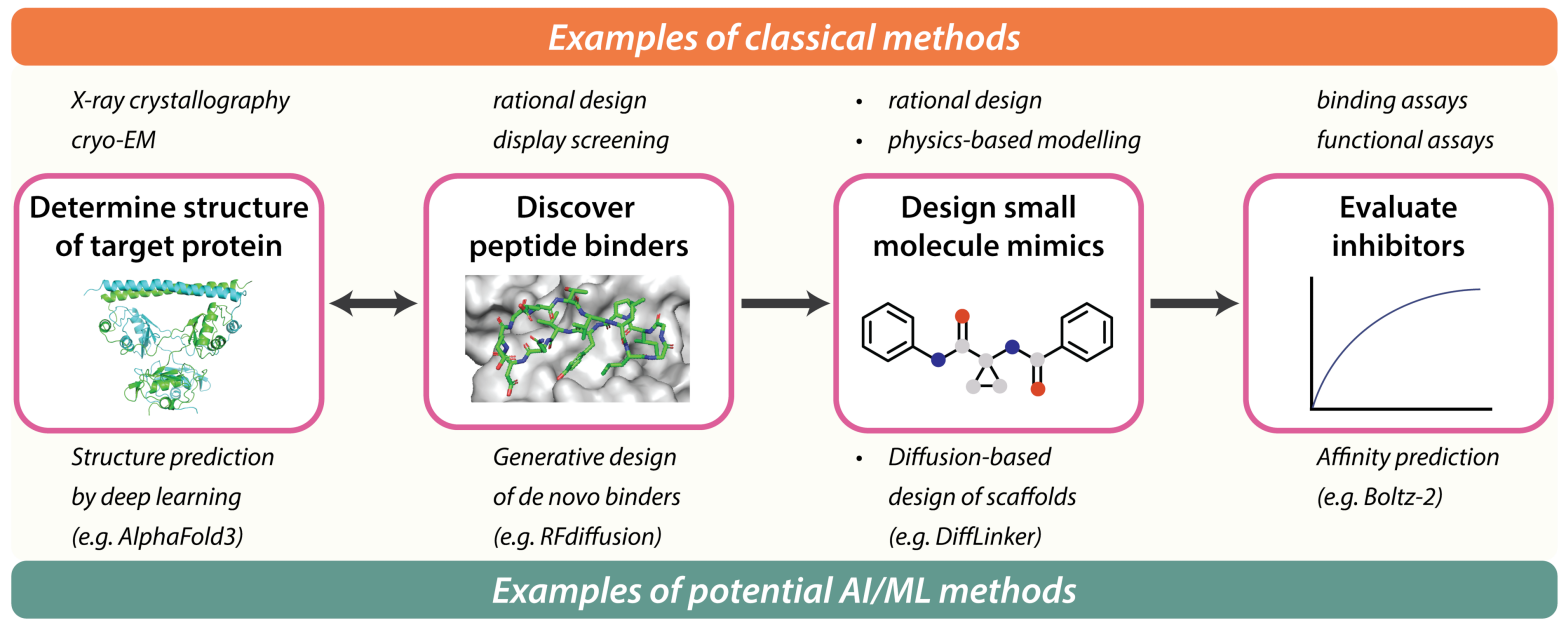
An example of a typical workflow from peptide hit discovery to small molecule evaluation. The top half shows typical methods used in each stage of the workflow, while the bottom half shows potential AI-based methods to augment the workflow. The left two images were generated using PyMOL (The PyMOL Molecular Graphics System, Version 3.1 Schrödinger, LLC.)

To understand where generative AI may play a role in transforming peptides into small molecules, we first briefly outline how traditional non-AI tools are typically used in the field. Starting from an experimental co-crystal structure of the bound complex, standard medicinal chemistry principles are used to probe structure–activity relationships (SAR) and determine the key interactions that form a minimal pharmacophore, supported by classical physics-based molecular modelling methods such as molecular dynamics (MD) and docking (Figure 1).

As an illustrative example, Yoshida and co-workers at Shionogi reported the design of small molecule inhibitors of nicotinamide *N*-methyltransferase [14] and β-herpesvirus proteases [15], starting from cyclic peptide hits obtained by mRNA display with flexible in vitro translation (also known as RaPID, random non-standard peptides integrated discovery [4]). Key interactions that comprise the pharmacophores were experimentally determined by alanine scanning and other amino acid substitutions to explore SAR, coupled with analysis of co-crystal structures obtained for the bound complexes, including computational analysis of the amino acid interactions (SiteMap [16]) and hydration in the binding pocket (grid inhomogeneous solvation theory [17] in AmberTools [18]). The pharmacophores were then used as the basis for virtual screening with docking (Glide [19–20]) to obtain candidate small molecules for experimental validation in functional assays.

#### Nascent examples of ML approaches

In contrast to physics-based models for extracting pharmacophores and predicting small molecule binders, ML methods are now emerging as alternatives for accomplishing these tasks.

An early application of ML for small molecule design based on peptide datasets was reported in 2023 by Hou and co-workers, where models were trained to classify ligands as ghrelin receptor binders [21]. The complete dataset for training and evaluation consisted of 548 peptides/peptidomimetics and 2193 small molecules spanning known binders, non-binders, and random compounds, each represented using one-hot encoding. A variety of well-established machine-learning classifiers were tested, including random forests, support vector machines, and gradient boosted decision trees. Strategies that used a larger proportion of the dataset for training showed reasonable predictive ability when evaluated on the remaining compounds as the validation set, as well as on an external set of 30 binders that was not in the original dataset. This result hints at the potential of ML models in predicting small molecule binders based on key peptide attributes, although the classifier was trained on more small molecules than peptides/peptidomimetics, making it unclear if peptide and peptidomimetic data alone would provide sufficient training to predict small molecule binders. The generality of this approach to other targets with no known small molecule binders has also yet to be evaluated.

Also in 2023, a deep learning-driven molecular generator for designing peptidomimetics was reported by Nakamura, Bajorath and co-workers [22]. Named DeepCubist, the method superimposes scaffolds from an sp^3^-rich skeleton library onto the target peptide to identify spatially aligned structures. Once a scaffold is selected, a transformer model converts the three-dimensional scaffolds into complete chemical structures by decorating the backbone with heteroatoms and unsaturated bonds. The resulting small molecules are designed to be synthetically tractable, although synthetic accessibility was noted to be somewhat restricted by the highly sp^3^-rich nature of the generated molecules. New iterations of these methods have since been reported by the authors [23], though experimental validation of predictions remains to be reported at the time of writing this Perspective.

### Opportunities for using generative AI and ML tools

Here, we highlight how multiple stages of the typical peptide to small molecule drug discovery workflow may be amenable to AI-enabled structure-based tools (Table 1). Starting from a target protein structure (which may be modelled by AI), generative models can be used to design de novo peptide binders. Upon identifying the minimal peptide pharmacophore, fragment-linking diffusion-based models can be used to recapitulate key binding features on a novel molecular scaffold. Subsequently, machine learning models can assist in triaging generated candidates prior to more resource-intensive modelling, laboratory synthesis, and evaluation in functional assays.

**Table 1:** Selected examples of structure-based AI/ML tools for potential end-to-end coverage of the peptide to small molecule pipeline. Tools are listed in alphabetical order. Year corresponds to the final publication date if peer-reviewed, or preprint date otherwise.

Capability	Tools	Developer

predicting structure of target protein (and complexes)	AlphaFold3 (2024) [25] Boltz-1 (2024) [26] Chai-1 (2024) [27] ESMFold (2023) [28] RoseTTAFold (2021) [29]	Google DeepMind MIT Jameel Clinic Chai Discovery Meta AI Institute for Protein Design, UW
generative design of peptide binders (including cyclic peptides)	AfCycDesign (2025) [30] BindCraft (2025) [31] BoltzGen (2025) [32] EvoBind2 (2025) [33] PepMimic (2025) [34] ProteinMPNN (2022) [35] RFpeptides (2025) [36]	Institute for Protein Design, UW Correia Lab, EPFL MIT Bryant Lab, Stockholm Ma Lab, Tsinghua University Institute for Protein Design, UW Institute for Protein Design, UW
fransforming peptides hits into small molecules	DiffLinker (2024) [37] Peptide2Mol (2025) [38] ShEPhERD (2024) [39]	Correia Lab, EPFL Ma Lab, Tsinghua University Coley Research Group, MIT
predicting affinities	Boltz-2 (2025) [40] RosettaVS (2024) [41]	MIT and Recursion Institute for Protein Design, UW

#### Deep learning for protein structure prediction

The growing wealth of structural data in the Protein Data Bank, along with ongoing improvements in computational processing power, has fuelled the success of protein structure prediction tools based on deep learning algorithms. Most notably, DeepMind’s AlphaFold2 heralded a breakthrough in protein structure prediction with its exemplary performance in the 14th Critical Assessment of protein Structure Prediction (CASP14) experiment in 2020 [24]. Since then, numerous models with advanced functionality have been released (Table 1), including AlphaFold3 which incorporates diffusion-based components, providing a range of options for predicting the structure of protein targets (including protein complexes and interactions with other biomolecules) where no experimental structure has been reported. While the accuracy of these models continues to increase with time, protein structures modelled on experimental data (X-ray crystallography, cryo-electron microscopy) still remain the gold standard as starting points for structure-based drug design.

#### Generative AI for peptide design

Building off the success of structure prediction tools, a major area of growth has been in new AI-based tools for designing de novo protein binders, many of which can be adapted to peptides. The release of RFdiffusion [42] and ProteinMPNN [35] from David Baker and co-workers at the Institute of Protein Design in 2022–23 popularised the use of diffusion models to build de novo protein backbones followed by sequence design. As with structure prediction, there has since been an explosion of new methods improving upon these initial methods (Table 1), including RFdiffusion2 [43] and RFdiffusion3 [44], along with integrated pipelines such as BindCraft [31] which are built upon tools such as ProteinMPNN and AlphaFold2. Other design tools that are specific for peptide binders also include the evolution-based EvoBind2 that only requires the protein target sequence as an input [33], and the diffusion-based PepMimic that uses structural information from binding interfaces [34]. Ongoing evaluation by independent groups suggests that reliable one-shot prediction of binders is currently target-dependent [45], and continued improvements may see these methods reach parity with traditional peptide discovery methods in the future.

#### Diffusion models to generate small molecule mimics

The reliable design of synthesisable small molecules that can accurately mimic a peptide pharmacophore remains the core challenge in the overall peptide to small molecule workflow. There are many AI tools for general ligand-based drug design under active development, spanning a range of generative architectures (e.g., variational autoencoders, generative adversarial networks). Here, we specifically highlight the potential of using diffusion-based models for generating small molecules that recapitulate key components of a peptide pharmacophore.

**Figure 2:**
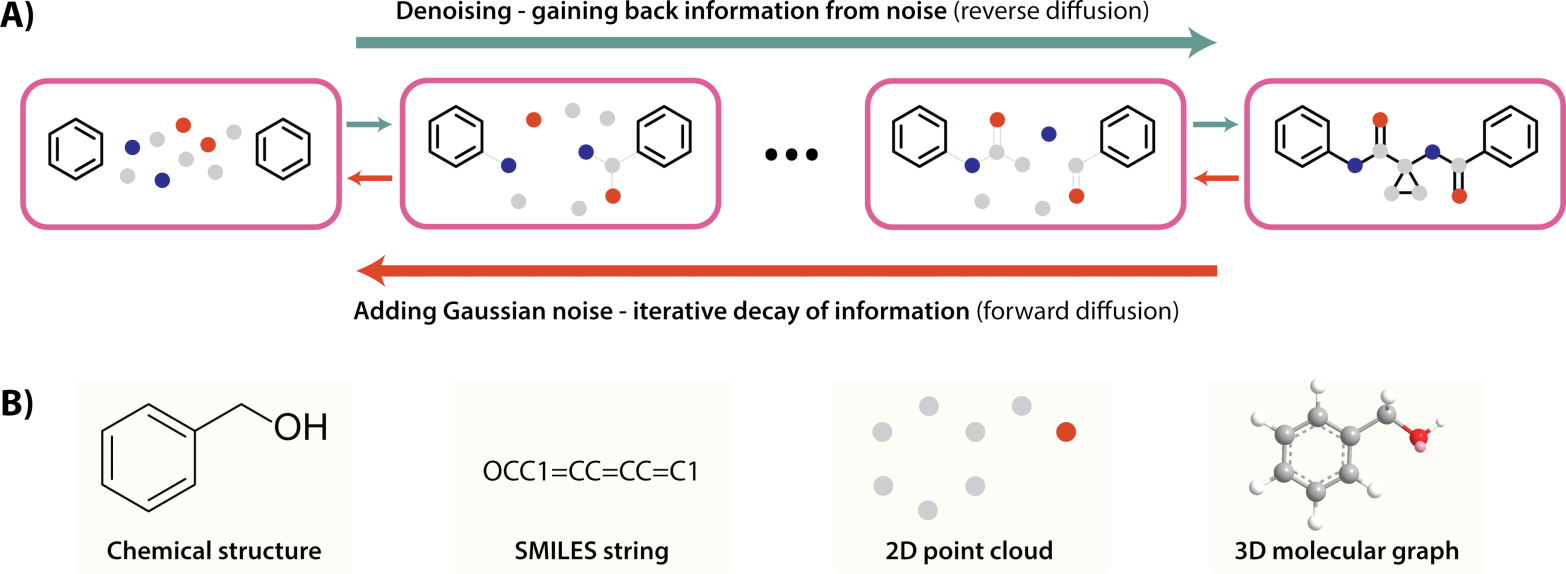
A) A non-technical schematic of a diffusion model, in which random noise is iteratively removed to reconstruct chemically valid molecules, based on training with a supplied dataset. B) Models can use different chemical representations as inputs, such as SMILES strings, point clouds, or molecular graphs.

In general, diffusion models function by progressively adding Gaussian noise to training data over a series of timesteps (forward diffusion). A neural network is then trained to reverse this process, iteratively reconstructing the original data structure through predicting and removing noise (reverse diffusion). Once trained, the model initialises and iteratively denoises random Gaussian noise to generate novel outputs (Figure 2a). Diffusion models have been particularly adept at generating new visual outputs such as images and videos, hence their suitability for de novo molecular design is not unexpected. In the case of small molecule design, the chemical inputs can be represented in different formats depending on the level of information and complexity required [46]. One-dimensional SMILES strings are the simplest form of molecular representation that lacks spatial information, while two- and three-dimensional molecular graph and point cloud representations are less compact but carry additional spatial information that is likely to be critical for small molecule binder design (Figure 2b).

In the context of transforming peptides into small molecules, diffusion models can be applied using a workflow reminiscent of fragment-based drug design. Starting from the key elements of a peptide pharmacophore (e.g., critical side-chains or backbone functional groups that interact with the target), diffusion models can generate candidate small molecules that spatially link or merge all these elements together. To preserve three-dimensional spatial information, the following examples of diffusion models all use three-dimensional atomic point clouds and an E(3)-equivariant (or SE(3)-equivariant) graph neural network for symmetry-consistent modelling of molecular structures [47].

DiffLinker is an example of a conditional diffusion model developed by Correia and co-workers that enables linking of multiple fragments in a predefined orientation [37]. After pretraining on linkers extracted from molecules originating from several compound databases (ZINC [48], CASF [49], GEOM [50]), input fragments were represented as three-dimensional point clouds, while the appropriately-sized linker was iteratively generated and refined by sampling atom types and positions using Gaussian noise as the starting point. The model could also be trained on a protein–ligand dataset (from Binding MOAD [51]) to factor in the constraints of typical binding pockets.

ShEPhERD is an interaction-aware diffusion model developed by Coley and co-workers that is capable of bioisosteric fragment merging [39]. For fragment merging, the model was trained on a subset from the MOSES database [52]. Then starting with a set of published fragment data from an experimental screen, ShEPhERD generated small molecule candidates with high similarity in electrostatic and pharmacophore scoring metrics.

Meanwhile, Peptide2Mol is a diffusion model developed by Ma and co-workers that leverages peptide-binder structural data to generate small molecules within the protein pocket. It is trained on a range of target-bound complexes, including small molecule ligands as well as peptides and protein binders [38]. Similar to DiffLinker, small molecule generation is initiated from Gaussian noise, then after iterative denoising within the target pocket, an additional pocket-aware refinement step is used to remove steric clashes and shape complementary issues.

Development of this class of generative molecular design tools is still in its infancy. Outputs typically require manual curation or integration with traditional physics-based molecular modelling to filter out unstable functional groups, strained rings and other unfavourable conformations. Experimental validation of predicted small molecules also remains to be conducted. Nevertheless, continued improvements in model design and quality input data may see these tools mature in future, potentially mirroring the trajectory of the aforementioned structure prediction and binder design tools.

#### Deep learning for predicting binding affinities

As a brief note, AI tools are also available for preliminary evaluation of small molecule candidates, providing potential alternatives to traditional computationally expensive physics-based workflows such as docking, MD, and free-energy perturbation (FEP) calculations for affinity estimation. For example, Boltz-2 features a strong emphasis on affinity prediction in addition to its core capability of structure prediction [40]. Other tools integrate AI methods into virtual screening pipelines, such as RosettaVS which trains a target-specific neural network during physics-based docking to prioritise candidates for more expensive computational modelling [41].

## Outlook

There is still significant progress that must be made before a complete AI-assisted computational workflow for transforming peptide hits into small molecule leads can be reliably achieved. While the early stages of structure prediction and peptide binder generation are rapidly maturing, small molecule design and evaluation methods are far less advanced, likely reflecting the more diverse and complex nature of small molecule chemical space, and the relatively sparse coverage of this space in training datasets. Nevertheless, early indications suggest that there could be a significant future role for generative AI in peptide to small molecule drug discovery workflows, even though ultimately, all predictions require validation in an experimental laboratory setting.

An open question is whether a peptide-first approach to drug discovery will continue to hold advantages in the future. Indeed, many target-based computational drug discovery tools that are ligand-agnostic are under development, along with small molecule prediction methods that do not require experimental data on target-specific peptide binders. Nevertheless, we envisage that given the ease of binder design and screening, synthetic accessibility, and in vitro assay validation, peptides will continue to play a significant role in supporting the small molecule drug discovery pipeline.

Just as the Critical Assessment of Structure Prediction (CASP) competition supported the development of protein structure prediction methods, the emergence of small molecule hit prediction competitions, such as the CACHE (Critical Assessment of Computational Hit-finding Experiments) challenge [53], are driving a pivotal shift toward rigorous, blind, and prospectively validated benchmarking. As methods improve, we expect such competitions to raise the bar and focus on increasingly challenging targets, such as protein–protein interactions without any known small molecule binders. Additionally, a focus on 'make-on-demand' constraints would allow generative models to move beyond the commercially available chemical space and into the realm of truly novel, de novo chemical space.

One potential barrier to rapid development and uptake of AI in drug discovery is the restricted accessibility of some models and datasets. While the development of proprietary AI-based platforms in private organisations such as large pharmaceutical companies and small start-up enterprises is driving local innovation within their respective organisations, such platforms are rarely shared for public use and may only be accessible through licensing at substantial cost. At the same time, one could argue that these platforms may have never been developed without the corporate structure that private enterprise offers. Although such debates are not new to drug discovery, we anticipate that the balance between proprietary and open-source development will have a disproportionate influence on the future of AI-based drug discovery, due to the data-intensive nature of the discipline.

Finally, if the full breadth of potential applications of AI in drug discovery come to fruition, one could imagine a fully-automated pipeline from target identification and virtual screening through to retrosynthetic planning, and robotics for hit-to-lead optimisation and functional evaluation in cellular models. The examples highlighted in this Perspective represent only a few of the many ongoing developments in the broader field. With the field moving at such a rapid pace, it is difficult to forecast which AI tools will become the next field standard akin to AlphaFold2 for structure prediction, and which others will fall short of more traditional computational or experimental methods. Only time will tell whether AI optimism in drug discovery is well-founded or not.

## Data Availability

Data sharing is not applicable as no new data was generated or analyzed in this study.
